# A recombinant chimeric protein specifically induces mutant KRAS degradation and potently inhibits pancreatic tumor growth

**DOI:** 10.18632/oncotarget.9996

**Published:** 2016-06-14

**Authors:** Ting Pan, Yiwen Zhang, Nan Zhou, Xin He, Cancan Chen, Liting Liang, Xiaobing Duan, Yingtong Lin, Kang Wu, Hui Zhang

**Affiliations:** ^1^ Institute of Human Virology, Sun Yat-Sen University, Guangzhou, Guangdong, China; ^2^ Key Laboratory of Tropical Diseases Control, Ministry of Education, Zhongshan School of Medicine, Sun Yat-Sen University, Guangzhou, Guangdong, China; ^3^ Guangdong Engineering Research Center for Antimicrobial Agent and Immunotechnology, Zhongshan School of Medicine, Sun Yat-Sen University, Guangzhou, Guangdong, China

**Keywords:** pancreatic cancer, Vif, Ras binding domain, ubiquitin, KRAS

## Abstract

Pancreatic cancer is one of the most lethal human diseases, with an all-stage 5-year survival rate below 5%. To date, no effective and specific therapy is available for this disease. Mutations in KRAS are frequently reported in pancreatic and many other cancers; thus, KRAS is an attractive therapeutic target. Our objective was to specifically eliminate mutant KRAS and induce cell death of tumors expressing this mutant protein. We thus constructed several chimeric proteins by connecting the C-terminal domains of several adaptor proteins of E3 ubiquitin ligases such as CBL, CHIP, E6AP, and VHL, as well as VIF encoded by human immunodeficiency virus type 1 (HIV-1), to the Ras binding domain (RBD) of Raf. Although all of these chimeric proteins caused the degradation of mutant KRAS and the death of KRAS-mutant-tumor cell lines, the RBD-VIF with a protein transduction domain (PTD), named PTD-RBD-VIF, had the strongest tumor-killing effect. Intraperitoneally administered recombinant PTD-RBD-VIF potently inhibited the growth of xenografted KRAS-mutant pancreatic cancer cells. Our findings indicate that recombinant PTD-RBD-VIF, a chimeric protein with a combined cellular-viral origin, could be further developed for the treatment of various tumors harboring mutant or over-activated KRAS, especially for cases presenting with pancreatic cancer recurrence after surgery.

## INTRODUCTION

Pancreatic carcinoma is an aggressive cancer and early diagnosis and radical surgery provide the only chance of long-term survival for patients [1–3]. A important feature of this cancer is that most pancreatic cancer cells harbor oncogenic mutations in the KRAS gene in the early stage, and the mutant KRAS is required to initiate pancreatic carcinoma [4, 5]. Substantial evidence has demonstrated the complexity of oncogenic KRAS signaling in promoting pancreatic cancer [6, 7]. Besides, many other tumors harbor mutations in the KRAS gene. In pancreatic cancer, colon cancer, and non-small cell lung cancer, the KRAS mutation rates are 90%, 45%, and 35%, respectively [8, 9]. Normal KRAS proteins function as molecular switches that cycle between the GDP-bound inactive and the GTP-bound active forms [10]. When bound to GTP, they interact with the downstream protein Raf and transduce the signal to activate various cell activities such as proliferation, differentiation, apoptosis, and migration [11, 12]. Specific point mutations in KRAS, especially those at position 12, maintain KRAS in its GTP-bound active form and consequently lead to tumor formation [13]. A variety of studies have been focused on the novel anti-kinase agents, which target the downstream kinases of KRAS signaling pathways such as MEK, PI3K and AKT [14–16]. Some of the inhibitors for these kinases are in clinical trials. However, the direct therapeutic agents for the inhibition of mutant KRAS is rare and urgently needed for the treatment of this disease.

Ubiquitination plays a critical role in numerous biological functions [17]. It involves three enzymes: E1, E2 and E3. Ubiquitin is activated by E1 and is transferred to the E2. E3 recognizes the protein substrates and brings them to E2, resulting in the ubiquitination of the target protein. Subsequently, the ubiquitinated protein is recognized and degraded by the 26S proteasome [18]. In humans, 2 E1s, approximately 50 E2s, and more than 600 E3s have been identified to date. E3s are categorized into 2 major types [19, 20]. One contains the HECT (Homologous to E6-AP C Terminus) domain and the other contains a RING (Really Interesting New Gene) finger domain or U-box domain, which is a modified RING motif without the full complement to Zn^2+^-binding ligands [19, 21]. These E3s act as adaptor molecules and participate in a variety of cellular functions [20]. Some E3s could be a part of fusion protein to retarget some important proteins and mediate their degradation [22–24]. Meanwhile, the application of the knockout system at protein level with an engineered E3 ubiquitin–protein ligase has been extensively explored [25–27].

The Ras binding domain (RBD) of Raf binds quite tightly to the GTP-bound form of Ras, whereas its affinity for Ras-GDP is three orders of magnitude lower [28, 29]. Therefore, the minimal RBD of Raf1 (aa 51–131) could serve as an activation-specific adaptor for mutant KRAS [30]. In this study, we screened a series of chimeric proteins by connecting the RBD domain with E3 adaptor proteins for specifically ubiquitinating and degrading mutant KRAS and found a novel therapeutic recombinant chimeric protein for targeting KRAS-mutant tumors, which can be particularly beneficial for the patients with pancreatic cancer.

## RESULTS

### Different chimeric proteins significantly inhibit the expression of mutant KRAS

**Figure 1:**
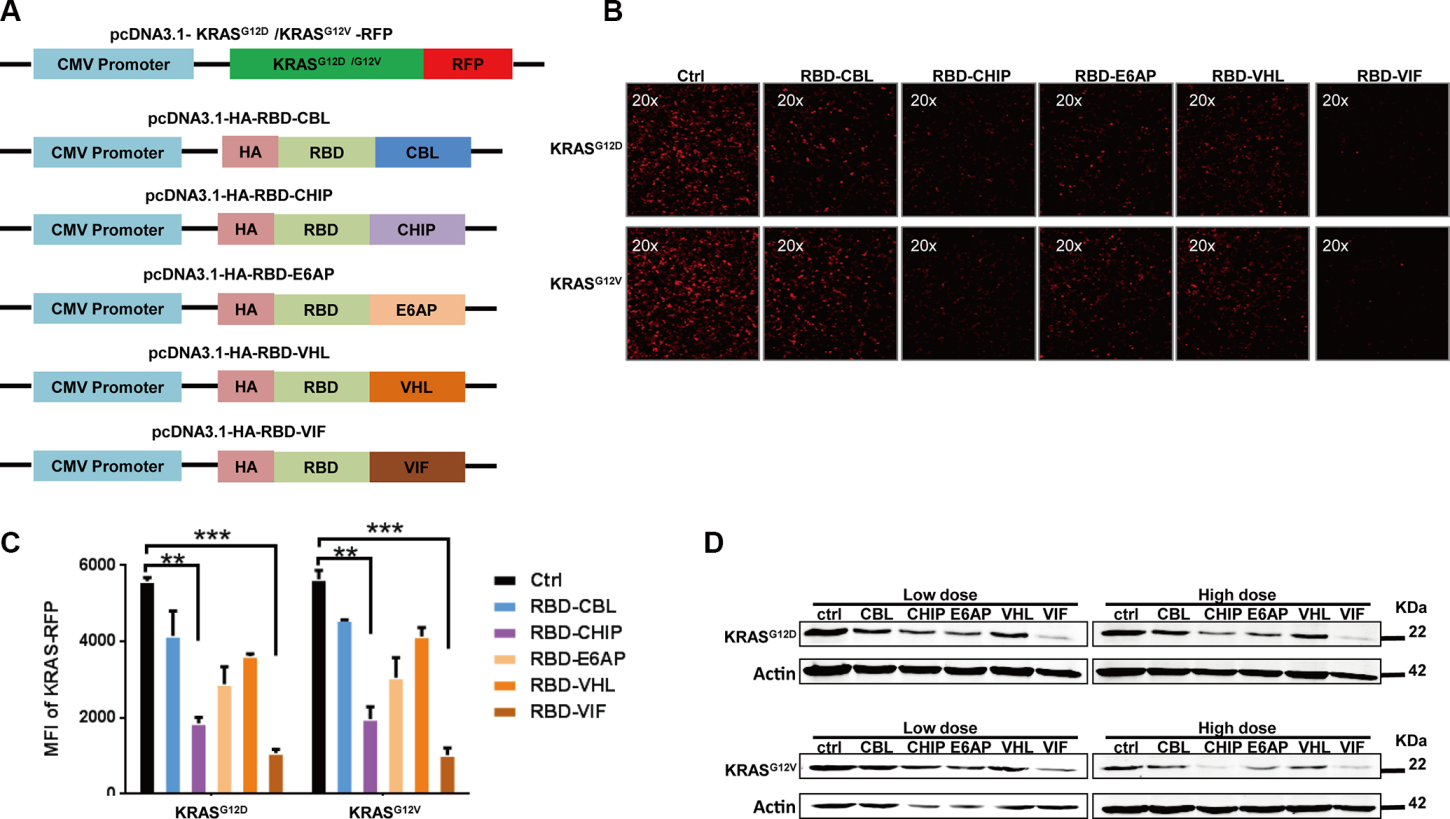
Different chimeric proteins significantly inhibit the expression of mutant KRAS^G12D^ or KRAS^G12V^ (**A**) Schematic of construction of different chimeric proteins and mutant KRAS-RFP-harboring plasmids. (**B**) Different plasmids harboring various chimeric proteins were co-transfected with KRAS^G12D^ or KRAS^G12V^ RFP-expressing plasmids into HEK293T cells. After 48 h, KRAS-RFP expression was detected under fluorescence microscope. (**C**) The MFI results were evaluated by flow cytometry and the mean ± SEM are shown. Error bars indicate SEM. *p < 0.05, **p < 0.01 compared to controls. (**D**) KRAS^G12D^ or KRAS^D12V^ RFP-expressing plasmids were co-transfected with high or low doses of adaptor plasmids into HEK293T cells, and the levels of mutant KRAS were determined by western blotting after 48 h.

In order to develop a therapeutic chimeric protein containing the RBD to knock down mutant KRAS, we selected several E3 ubiquitin ligases or adaptors. These included the ubiquitin ligase E6 associated protein (E6AP) encoded by the UBE3A gene, which belongs to HECT family and interacts with the E6 protein of human papillomavirus types 16 and 18, resulting in ubiquitination and proteolysis of tumor protein p53 [31]; Casitas B-lineage lymphoma proto-oncogene (CBL), a proto-oncogene that encodes a RING finger E3 ligase [32]; and carboxyl-terminus heat shock cognate 70-interacting protein (CHIP), with a C-terminal U-box domain, which is known to ubiquitinate short-lived proteins [33]. Of the well-known adaptors of E3 ligase, we selected von Hippel-Lindau syndrome (VHL) and HIV-1 virion infectivity factor (VIF), which are adaptors for the complex including elongin B, elongin C, and cullin-2/5 that possesses E3 ubiquitin ligase activity [34, 35]. The protein levels of these various chimeric plasmids were confirmed by western blot. As shown in Figure 1A, the RBD of Raf-1, which has been reported to bind to mutant KRAS, was chosen as the KRAS-binding domain for the chimeric proteins. To determine whether these chimeric proteins could degrade mutant KRAS, a plasmid encoding mutant KRAS-RFP fusion protein was constructed and used for quantification of the KRAS level in HEK293T cells. After co-transfection of the mutant KRAS-RFP-harboring plasmids and the plasmids encoding various chimeric proteins, we found that the cellular KRAS-RFP levels, determined by fluorescence microscopy, were significantly decreased by these chimeric proteins, especially by RBD-CHIP and RBD-VIF (Figure 1B). The levels measured by mean fluorescent intensity (MFI) also showed similar results (Figure 1C). Western blotting revealed that the expression of mutant KRAS^G12D^ or KRAS^G12V^ was also potently inhibited by these chimeric proteins in a dose-dependent manner (Figure 1D). Besides, the chimeric RBD-VIF have no effect on the degradation of wild-type KRAS (Supplementary Figure S1).

### Recombinant chimeric proteins induce cell death in mutant KRAS-expressing tumor cell lines

**Figure 2:**
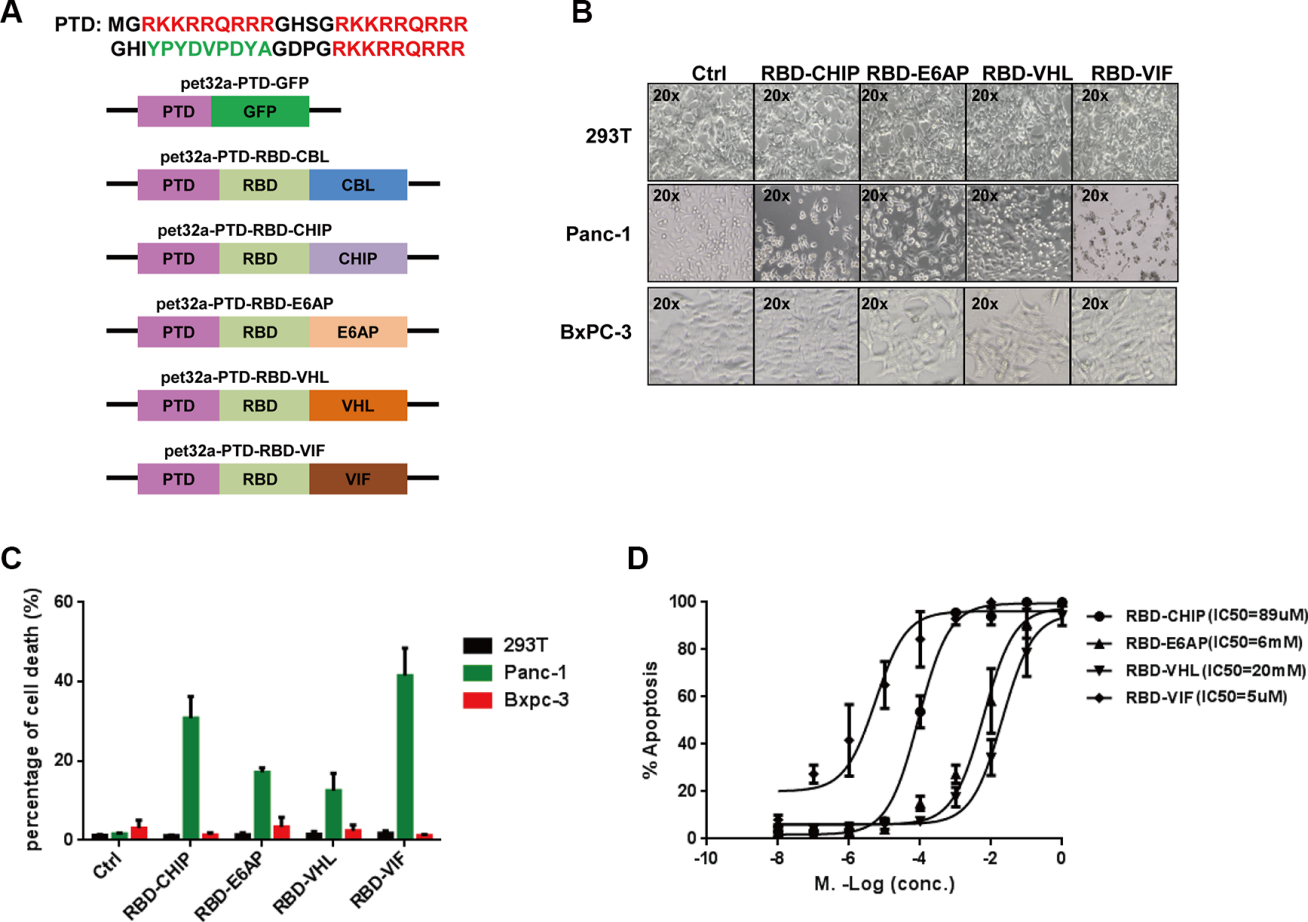
Purified chimeric proteins induce cell death in tumor cell lines (**A**) Schematic of construction of different chimeric proteins. (**B**) The purified proteins were added to different cell cultures. After 48 h, cells were examined under microscope. (**C**) The percentage of cell death was evaluated by MTT assay, and mean ± SEM is shown. Error bars indicate SEM. (**D**) Different doses of chimeric proteins were added to Panc-1 cells and IC50 was calculated. Error bars indicate SEM.

We then examined the specific degradation of mutant KRAS by the recombinant chimeric proteins. To facilitate the uptake of recombinant chimeric proteins into cells, the three repeated protein transduction domains (PTDs) of HIV-1 Tat protein, which have powerful transmembrane transporting capabilities [36], were connected to the N-termini of the chimeric proteins (Figure 2A). As the expression of recombinant PTD-RBD-CBL was not induced well by 1 mM isopropylthio-β-d-galactoside (IPTG), we did not continue to test this chimeric protein. After expression and purification of the rest of the recombinant chimeric proteins, we examined their abilities to induce cell death in several mutant KRAS-expressing tumor cell lines. We found that almost all of the cells harboring KRAS-mutant died at 48 h after treatment with the recombinant chimeric proteins, whereas the growth of non-KRAS mutant cell lines, such as HEK293T cells and Bxpc-3 cells, were not inhibited. Quantification of cell death showed the similar results (Figure 2B and Supplementary Figure S2). PTD-RBD-VIF and PTD-RBD-CHIP exhibited high efficiency to specifically induce the death of Panc-1 cells (Figure 2C). Furthermore, the IC50 of PTD-RBD-CHIP, PTD-RBD-E6AP, PTD-RBD-VHL and PTD-RBD-VIF in the Panc-1 cell line was 89 μM, 6 mM, 20 mM and 5 μM, respectively, as measured by flow cytometry. Thus, PTD-RBD-VIF showed the best capacity to induce cell death (Figure 2D).

### Evaluation of immunogenicity and safety of purified proteins

**Figure 3:**
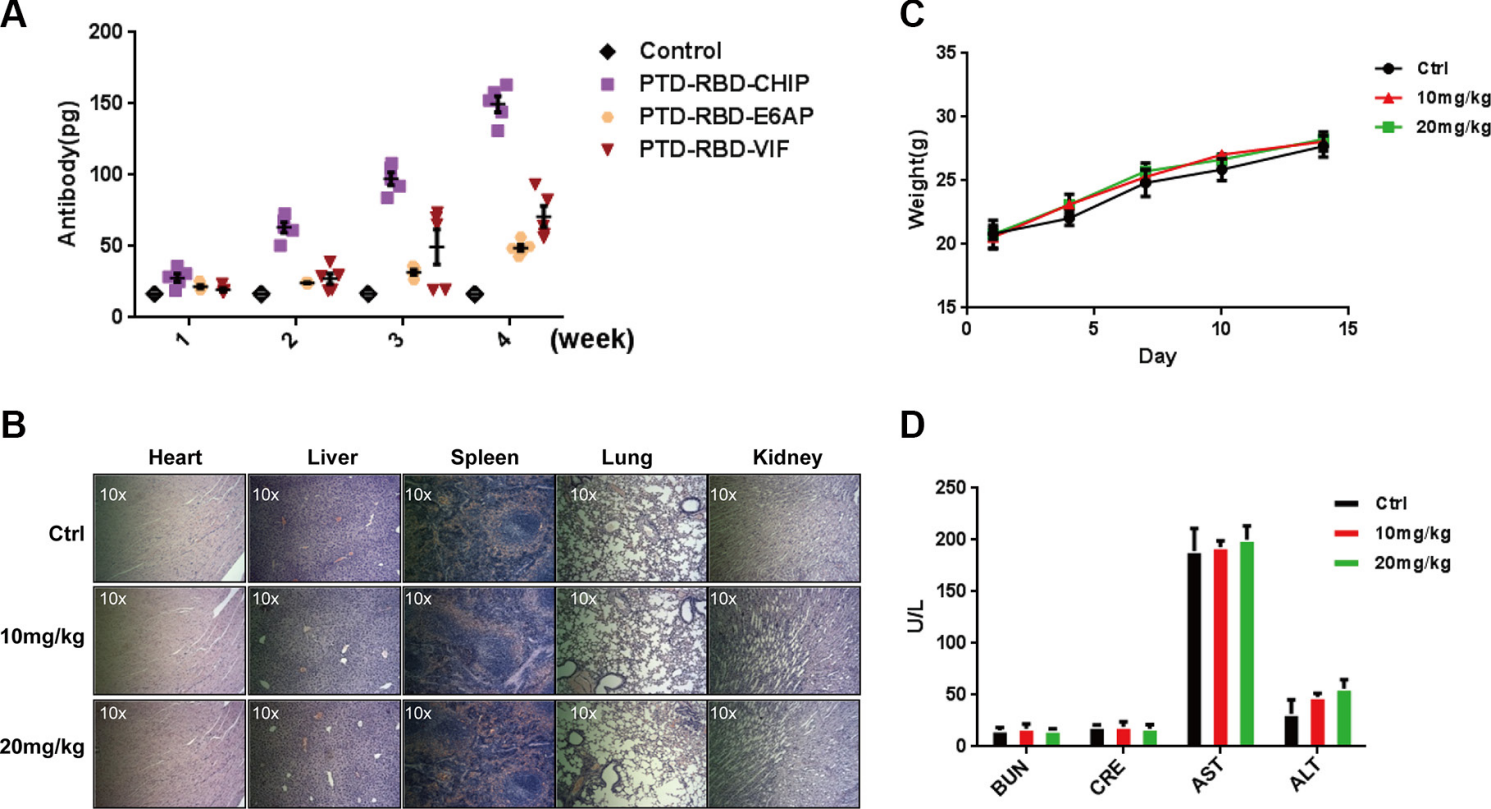
Purified PTD-RBD-VIF has low immunogenicity and toxicity (**A**) Different chimeric proteins (PTD-RBD-CHIP, PTD-RBD-E6AP, PTD-RBD-VIF) were intraperitoneally injected into 6 mice individually and the immunogenicity of each protein was detected by ELISA after 4 wk. Error bars indicate SEM. (**B–D**) The safety of PTD-RBD-VIF was examined by the acute toxicity test. (B) Vital organs, including spleen, lung, liver, kidney, and heart, from mice treated with various doses of PTD-RBD-VIF were histologically sectioned and stained with HE. (C) Weight of male BALB/c mice after intraperitoneal injection of PTD-RBD-VIF. Error bars indicate SEM. (D) The effect of PTD-RBD-VIF on the hepatic and renal functions of mice. Error bars indicate SEM. (BUN: Blood urea nitrogen, CRE: Creatine, AST: Aspartate transaminase, ALT: Alanine transaminase).

Considering the possible development of protein inhibitors for pancreatic cancer, it is important to determine the immunogenicity and safety of the recombinant chimeric proteins. To this end, 4-week-old female BALB/c mice were immunized subcutaneously with 1 μg of PTD-RBD-VIF or PTD-RBD-CHIP. PTD-RBD-VIF exhibited considerably low immunogenicity (Figure 3A). To determine the toxicity, the acute toxicities of PTD-RBD-VIF on mice were tested. Two weeks after intraperitoneal injection of PTD-RBD-VIF at 0 mg/kg, 10 mg/kg, or 20 mg/kg, 5 male BALB/c mice did not show significant histological changes in heart, liver, lung, spleen, and kidney in HE staining (Figure 3B). Additionally, no significant abnormality was found in body weight (Figure 3C). The functions of key enzymes, including AST, ALT, BUN, and CRE, in the histological sections of these organs were also normal ranges (Figure 3D). Collectively, recombinant PTD-RBD-VIF had the highest efficiency to specifically induce the degradation of mutant KRAS and the death of cells harboring mutant KRAS, with low immunogenicity and tolerable toxicity in mice.

### RBD-VIF mediates the ubiquitination and degradation of mutant KRAS and inhibits the downstream of MAPK-ERK pathway

**Figure 4:**
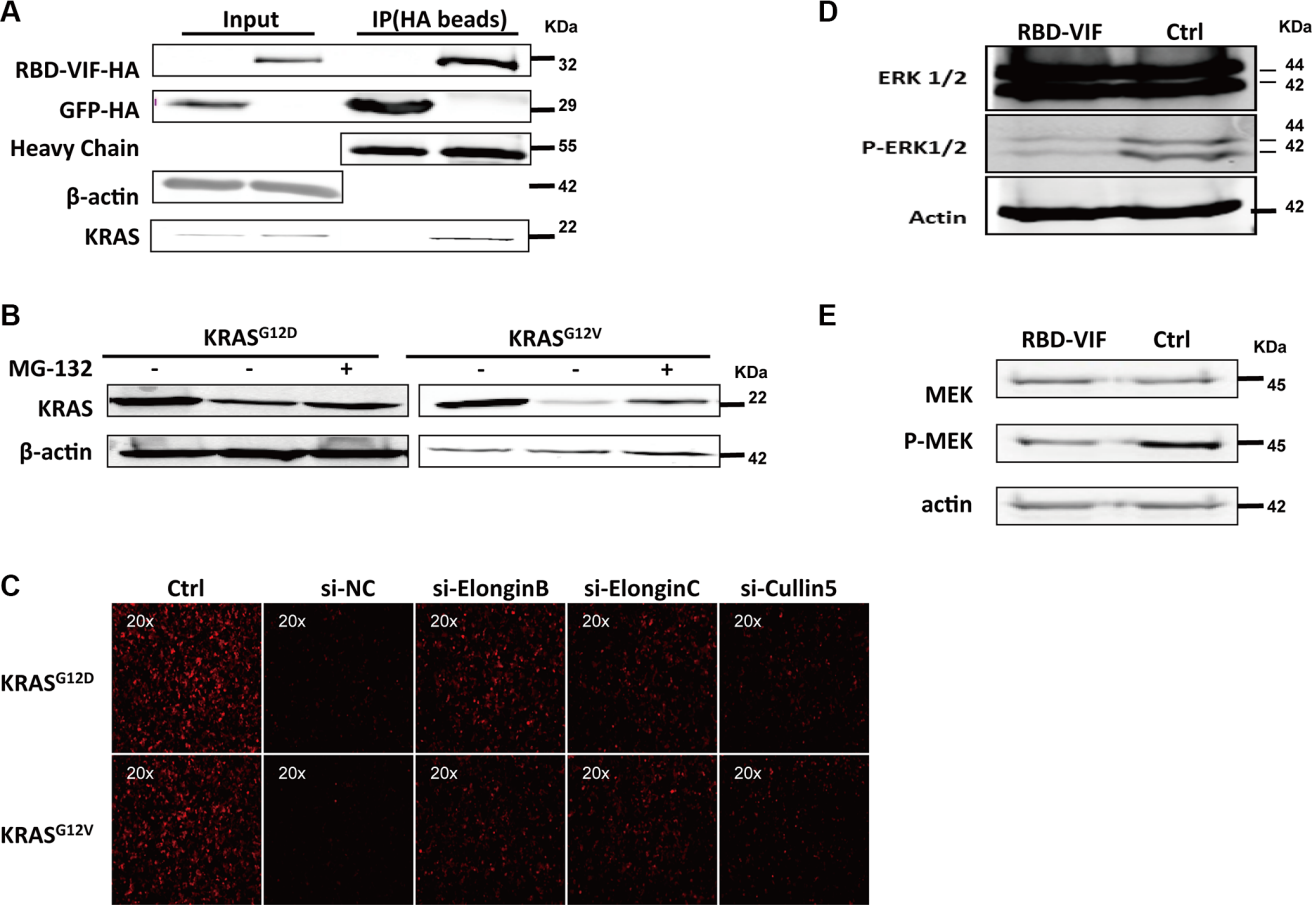
RBD-VIF mediates the degradation of mutant KRAS through Vif-mediated ubiquitin system and inhibits the downstream of MAPK-ERK pathway (**A**) RBD-VIF-HA plasmid and KRAS plasmid were co-transfected into HEK293T cells, and 48 h later, cells were collected for co-immunoprecipitation experiments. GFP-HA plasmid was transfected as a negative control. (**B**) RBD-VIF-HA plasmid and mutant KRAS plasmid were co-transfected into HEK293T cells and the culture was maintained for 12 h with or without 2 *μ*M MG-132 before harvesting the cells for western blot analysis at 48 h. (**C**) Mutant KRAS-RFP-expressing plasmids were co-transfected with si-NC, si-Elongin B, si-Elongin C, or si-Cullin 5 respectively into HEK293T cells. After 48 h, the expression of KRAS-RFP was detected under fluorescence microscope. (**D**) Panc-1 cells were treated with RBD-VIF or a control protein (PTD-GFP). After 24 h, cells were harvested for western blot analysis to detect the phosphorylation of ERK1/2. (**E**) Panc-1 cells were treated with RBD-VIF or the control protein PTD-GFP for 24 h and then the cells were harvested for western blot to detect P-MEK expression.

To investigate the mechanism underlying the specific degradation of KRAS by RBD-VIF, the HA-tagged RBD-VIF and mutant KRAS plasmids were co-transfected into HEK293T cells and the interaction between mutant KRAS and RBD-VIF was examined. Mutant KRAS was co-immunoprecipitated with HA-tagged RBD-VIF chimeric protein (Figure 4A). To further confirm the involvement of the ubiquitin system in RBD-VIF-mediated KRAS degradation, we treated the transfected Panc-1 cells with MG-132, a specific inhibitor of the 26S proteasome. The levels of mutant KRAS were restored in the presence of MG-132 (Figure 4B). It is known that Vif binds to Elongin B, Elongin C, and Cullin5 to form an E3 ubiquitin ligase complex resulting in the ubiquitination of APOBEC3G [34]. All of the protein members of this complex are vital for its function. To determine whether or not these proteins are involved in the degradation of mutant KRAS, we knocked down the expression of Elongin B, Elongin C, and Cullin 5 using specific siRNAs. The levels of mutant KRAS-RFP were rescued by the suppression of these proteins (Figure 4C), supporting that RBD-VIF mediates the degradation of mutant KRAS through the Vif-conjugated ubiquitin system. Alternatively, because ERK1/2 and MEK are major downstream kinases in the KRAS signaling pathway [37, 38], we measured the level of ERK1/2 and MEK phosphorylation to explore the mechanism by which RBD-VIF suppresses mutant KRAS. As shown in Figure 4D and 4E, treatment of Panc-1 cells with recombinant PTD-RBD-VIF significantly decreased the phosphorylation of ERK1/2 and MEK. These results were consistent with a previous report that inhibition of KRAS inhibits the Raf/MEK/ERK signaling pathway [38].

### Different PTD-RBD-VIF administration routes for treatment of the xenograft tumors in nude mice

**Figure 5:**
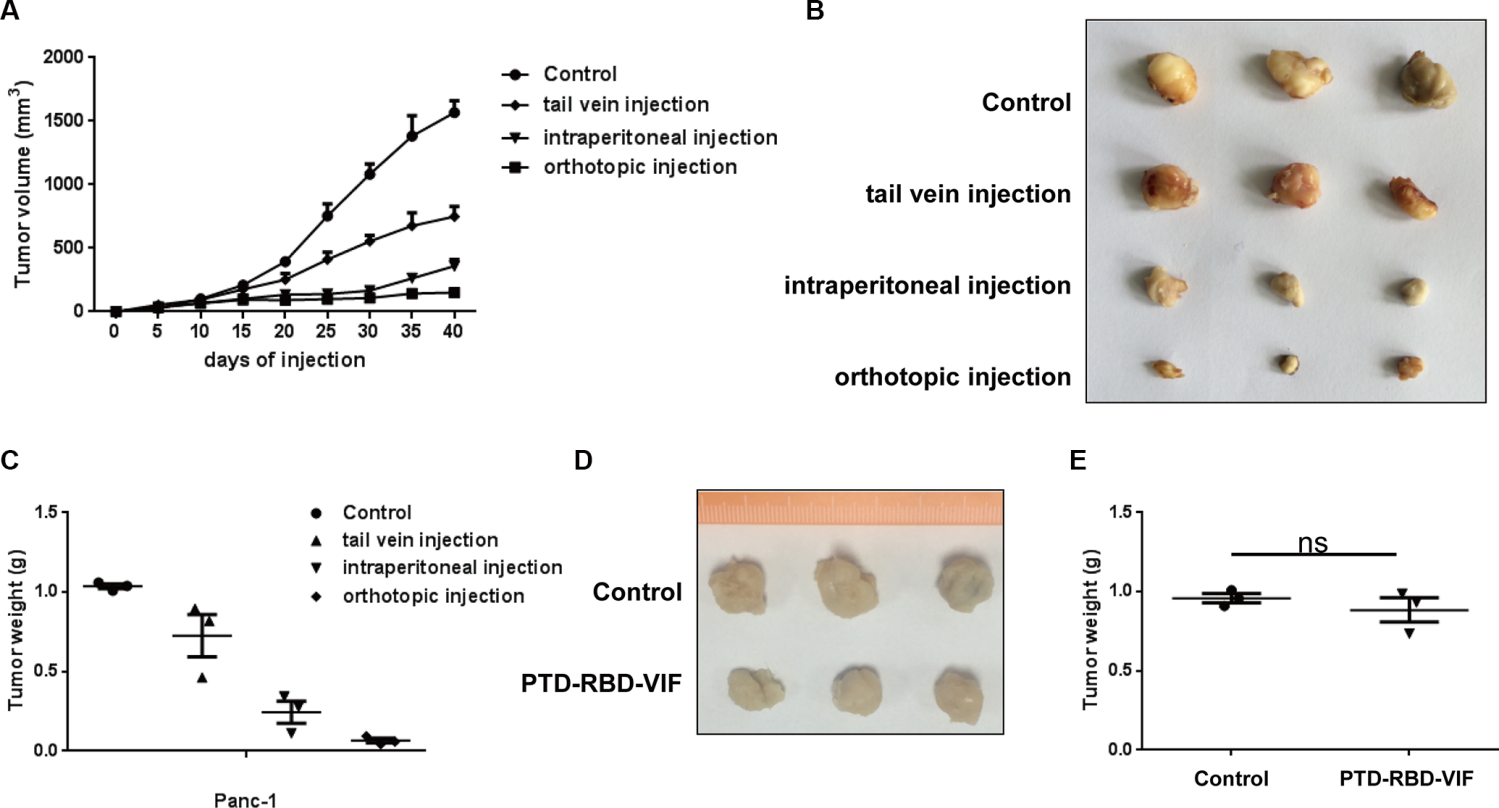
Comparison of PTD-RBD-VIF with different ways of administrations in nude mice (**A–C**) Xenografts were established using Panc-1 cell line in female BALB/c nude mice. Four groups were randomly assigned and injected with control protein or recombinant PTD-RBD-VIF by tail vein injection, intraperitoneal injection and orthotopic injection every 3 days (*n* = 3). (A) Tumor volumes were measured every 5 days. Error bars indicate SEM. (B) Tumor formation assay to evaluate the effect of different PTD-RBD-VIF delivery pathways. (C) The data represent mean ± SEM (*n* = 3). Error bars indicate SEM. (**D–E**) Xenografts were established using BxPC-3 cell line in female BALB/c nude mice. Two groups were randomly assigned and injected with control protein or recombinant PTD-RBD-VIF by tail vein injection every 3 days (*n* = 3). (D) Tumor formation assay to evaluate the effect of different PTD-RBD-VIF delivery pathways. (E) The data represent mean ± SEM (*n* = 3). Error bars indicate SEM.

To further study the *in vivo* anti-tumor activity of recombinant PTD-RBD-VIF, we established a Panc-1 cell xenograft tumor model in BALB/c nude mice. Each mouse was subcutaneously injected with 1 × 10^6^ Panc-1 cells at the abdomen and randomly divided into 4 groups. The mice in the experimental groups were injected with 100 mg of PTD-RBD-VIF every 3 days. The control protein, recombinant PTD-GFP, was injected into the mice in control group. As shown in Figure 5A–5C, the orthotopic injection was more effective than tail vein injection and intraperitoneal injection in this xenografted mouse model. Furthermore, we also established a xenograft tumor model in BALB/c nude mice with BxPC-3 cells. The mice were injected with 1 × 10^6^ BxPC-3 cells at the abdomen and orthotopically treated with PTD-RBD-VIF or a control protein. As expected, PTD-RBD-VIF has no effect on pancreatic cell line without mutant KRAS (Figure 5D and 5E).

### Recombinant PTD-RBD-VIF protein induces tumor cell death *in vivo*

**Figure 6:**
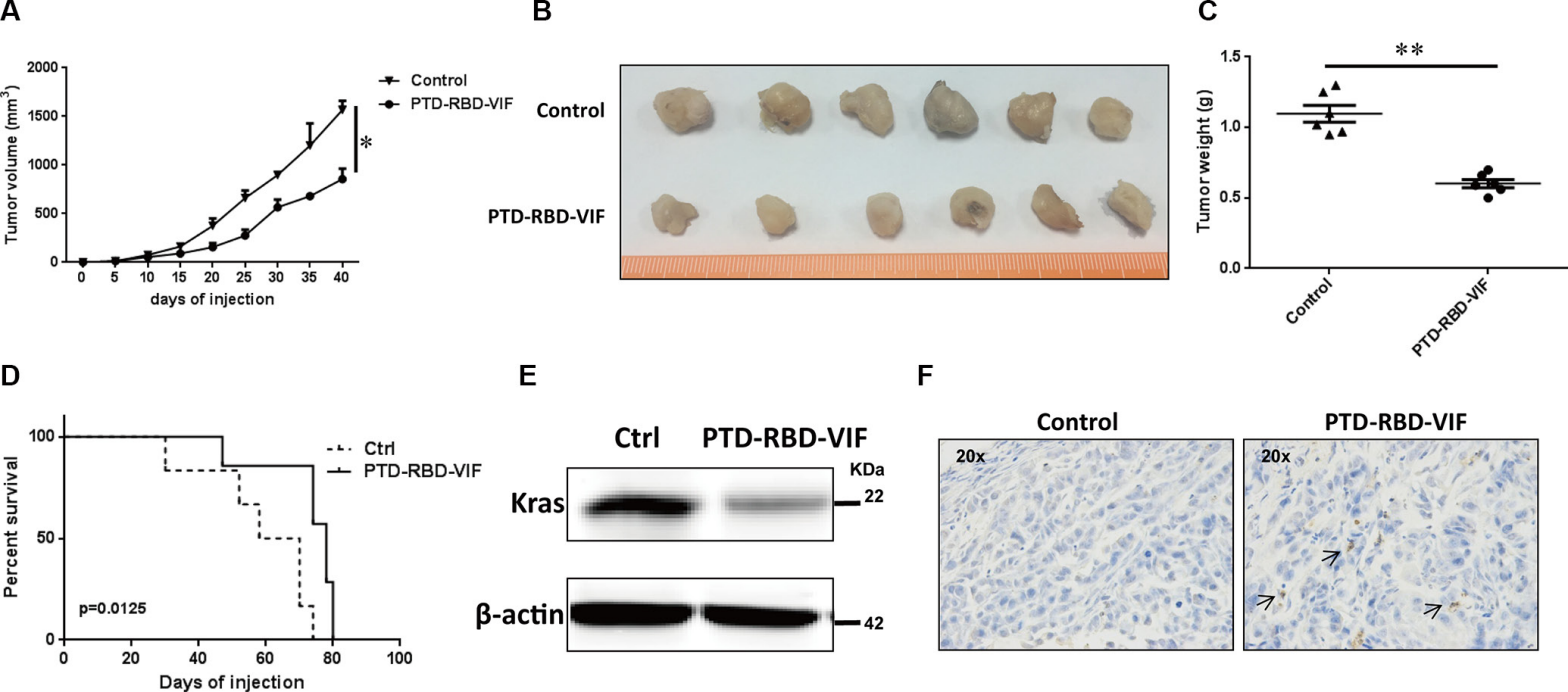
PTD-RBD-VIF potently inhibits pancreatic tumor growth with intraperitoneal injection *in vivo* Xenografts were established using Panc-1 cell line in female BALB/c nude mice. Two groups were randomly assigned and injected (i.p.) with recombinant PTD-RBD-VIF or control protein every 3 days (*n* = 6). (**A**) Tumor volumes were measured every 5 days. Error bars indicate SEM. (**B**) Tumor formation assay to evaluate the effect of PTD-RBD-VIF or control protein. (**C**) The data represent mean ± SEM (*n* = 6). Error bars indicate SEM. (**D**) Survival curve for 2 groups of mice. (**E**) Representative western blot to detect the expression of KRAS in the tumors in these 2 groups. (**F**) Representative TUNEL staining of tumors in each group.

Considering the anatomical location of pancreas and the actual clinical treatments for pancreatic cancer, we continued our study by using intraperitoneal injection. Again, the mice were injected intraperitoneally with 100 μg of PTD-RBD-VIF or control protein PTD-GFP every 3 days. A significant difference in tumor volume was observed between the PTD-RBD-VIF–treated and control groups at days 20 to 40 after treatment (Figure 6A). The representative gross morphology of these tumors is shown in Figure 6B. Tumor weight and the survival rate of mice in the 2 groups were significantly different (Figure 6C and 6D). Furthermore, to confirm that PTD-RBD-VIF inhibited the expression of mutant KRAS *in vivo*, representative tumor tissues were digested with collagenase and hyaluronidase and cells were disrupted and analyzed by western blotting. The expression of mutant KRA was indeed decreased (Figure 6E). TUNEL staining revealed that cell death was increased in the RBD-VIF treated group compared to the control group (Figure 6F). Taken together, our data indicate that recombinant PTD-RBD-VIF interacts with mutant KRAS and induces its ubiquitination and degradation, thereby inhibiting the growth of pancreatic cancer cells *in vitro* and *in vivo*.

## DISCUSSION

KRAS has long been considered a target for cancer therapeutics. The siRNA-driven knockdown of KRAS was previously shown to cause apoptosis of KRAS mutant-expressing cancer cells [39, 40]. Although RNA interference (RNAi) provides an alternative therapeutic approach for inhibiting KRAS gene function, the efficientdelivery of siRNAs remains a problem and early attempts to develop small molecules targeting KRAS as a direct pancreatic cancer therapy were not successful. Several drugs have been developed to inhibit the farnesylation of RAS, is essential for the biological activity of RAS [41, 42]. A farnesylation inhibitor, R115777, has completed a Phase II clinical trial for use in the treatment of pancreatic cancer. Another farnesylation inhibitor, anthroquinonol, is in a phase II clinical trial for non-small cell lung cancer (NSCLC). In addition, a recent report showed that an engineered ubiquitin ligase suppressed pancreatic cancer cells by targeting mutant KRAS [23]. However, these chimeric genes were carried by a lentiviral vector. Given the low delivery efficiency of recombinant lentiviral viruses into the tumor mass, this gene therapy strategy is unlikely to be further developed as a new treatment modality in the near future.

In this study, we generated a novel recombinant chimeric protein PTD-RBD-VIF for specific ubiquitination and degradation of mutant KRAS. HIV-1 Vif is a highly conserved viral protein that induces ubiquitination and degradation of host restriction factor APOBEC3G [34, 43, 44]. By comparative analysis, we found that PTD-RBD-VIF is the most effective of the chimeric proteins for degradation of mutated KRAS. PTD-RBD-VIF degrades KRAS through the same pathway used by VIF to degrade APOBEC3G. This was consistent with the previous finding that depletion of elongin B, elongin C, or Cullin5, which are components of the E3 ligase complex required for the degradation of APOBEC3G, decreased the degradation of mutant KRAS [34]. Although it has been reported that several other engineered E3 ligases could dregade oncoproteins such as HER2, C-MYC, and KRAS, none of them has been further developed as an effective recombinant protein for the treatment of cancer [22–24]. It could be due to their relatively low efficiency to induce the cancer cell death, as we have demonstrated in Figure 2D. Our study indicates that the IC50 of recombinant PTD-RBD-VIF to induce cell death is 5 uM, which is much lower than that of other chimeric proteins. We are not surprised by this result because the highly-conserved nature of Vif suggests that its ubiquitination function is critical for HIV-1.

Pancreatic cancer is the fourth leading cause of cancer-related deaths and the usual survival rate of patients is under six months [3]. Currently, the routine treatment includes surgery, chemotherapy, radiotherapy, and administration of immunosuppressants [45, 46]. Even when the pancreatic tumor is surgically resected, the post-surgery survival rate is exceptionally poor. Recurrent pancreatic carcinoma remains a significant therapeutic challenge and no treatment has shown a strong impact to date [47]. Because pancreatic carcinoma recurrences after surgery resecting are mainly local retroperitoneal recurrence, quickly followed by hepatic metastases or peritoneal dissemination, it is difficult to choose secondary surgery [48, 49]. Given the complex biology and clinical characteristics of pancreatic cancer, we designed an intraperitoneal injection to simulate the natural clinical condition, although the orthotopic injection was more effective than tail vein injection and intraperitoneal injection in our xenografted mouse model of pancreatic cancer. After direct injection of recombinant PTD-RBD-Vif into the abdominal cavity, the chimeric protein potently inhibited the growth of pancreatic cancer implanted subcutaneously at the abdomen, which mimicked the retroperitoneally-located pancreatic cancer. Furthermore, intraperitoneal administration of this recombinant protein was found to be safe and convenient. Our findings reveal that the recombinant PTD-RBD-VIF has potential for treatment of pancreatic carcinoma *in vivo*. We expect that intraperitoneal administration of this recombinant protein after surgery resecting pancreatic cancer could increase the survival rate and quality of life of pancreatic carcinoma patients, although further evaluation of its druggability is needed.

## MATERIALS AND METHODS

### Cell culture and transfection

Human Panc-1 cells, Bxpc-3 cells and HEK293T cells were obtained from ATCC and grown at 37°C in Dulbecco's modified Eagle's medium (Gibco) supplemented with 10% fetal calf serum (Gibco), 100 units/ml of penicillin and 100 μg/ml of streptomycin (Gibco). Lipofectamine 2000 (Invitrogen) was used for transfection of plasmids or si-RNAs respectively by following the instruction of manufacturer.

### Plasmid construction

The wide-type KRAS DNA fragment was generated by PCR with the isolated genomic DNA of Bxpc-3 cells as the template. It was then inserted into pcDNA3.1 vectors. The G12D and G12V mutants were generated by point mutation assay, as described by us previously [50]. The sequence of Vif C-terminus was amplified with PCR from an infectious HIV-1 clone pNL4-3 and inserted into pcDNA3.1 harboring an intron. The other adaptor genes, CHIP, E6AP, CBL, VHL, were amplified from cDNA of HEK293T cells by PCR. The accuracy of all the clones was confirmed by DNA sequencing.

### Small interfering RNA (siRNA) knockdown

The human Elongin B, Elongin C and Cullin5-specific siRNAs and a negative control siRNA were purchased from Ribo, Inc. (Guangzhou, China). The HEK293T cells were transfected with 50 nM siRNA using Lipofectamine RNAimax (Invitrogen, Inc.). After 48 h, the knockdown efficiency were detected by qRT-PCR.

### Protein purification and endotoxin removal

The plasmids pET32a harboring PTD-RBD-VIF or other chimeric genes were transformed into E. Coli BL21 competent cells (Novagen) respectively. After the expression of proteins was induced by 1 mM isopropylthio-β-d-galactoside, the bacterial cells were lysed by sonication. The insoluble fraction was pelleted at 10,000 × g for 10 min, and the supernatant was applied to a Ni-conjugated agarose bead column (GE). After washing, the bound His fusion proteins were eluted with 500 μM Imidazole. The proteins were then suspended in PBS buffer and the concentration was measured by the Bradford method. For the *in vivo* tumor model experiments, the His-tag was removed first. And then the no-tagged PTD-RBD-VIF proteins was pruified by a heparin-agarose chromatography and subsequently ion exchange [51]. The proteins were then suspended in PBS buffer and the concentration was measured by the Bradford method. The purities of expressed proteins were > 95%. Moreover, the possible endotoxin had been removed from the recombinant protein with Triton X-114 as described previously and the residual endotoxin was examined with limulus amoebocyte lysate (LAL) assay (Cambrex Bio Science) by following the instruction of manufacturer [52]. The samples were then aliquoted and frozen at −80°C.

### Immunogenicity assay in mice

All animal works were performed incompliance with the institutional guidelines and approved protocols. The 4-week-old female BALB/c mice were intradermally immunized with the recombinant PTD-RBD-VIF or PTD-RBD-CHIP protein mixed in a complete Freund's adjuvant (Sigma) at day 0. Then these mice were boosted three times with proteins mixed in an incomplete Freund's adjuvant (Sigma) every week. Protein-specific antibody responses were measured by ELISA. Briefly, the recombinant PTD-RBD-VIF or PTD-RBD-CHIP proteins diluted in PBS were coated in 96-well plates overnight at 4°C, followed by 30 min of blocking with non-fat milk. The serum samples were then added and incubated at room temperature for 1 h. After washing, the bound antibodies were detected using the HRP-labeled goat anti-mouse IgG (H +L) and TBM substrate. The OD 450 nm was recorded and used as a relative measurement for antibody titer.

### Western blot and co-immunoprecipitation (Co-IP)

HEK293T cells were transfected with chimeric adaptor-expressing plasmids and mutant KRAS-expressing plasmid and were lysed 48 h later with lysis buffer. The lysate were then subjected to electrophoresis, followed by transferring onto the membrane and detection with the primary antibodies including anti-KRAS (mouse monoclonal, CST) or anti-beta-actin (mouse monoclonal, MBL). For the co-IP experiment, the lysate were incubated with anti-HA beads (Sigma) overnight at 4°C. Then IP products were centrifuged and washed three times with lysis buffer. Western blot was conducted to analyze the immunoprecipitated samples with the primary antibodies including anti-KRAS (rabbit polyclonal, MBL) or anti-HA (mouse monoclonal, MBL).

### Cell toxicity test

Cell toxicity assay was performed with the CellTiter-Glo Luminescent Cell Viability Assay Kit (Promega). The instructions of manufacturer were followed. Luminescence was recorded with a Promega plate reader.

### Apoptosis assay

An Annexin V-FITC Apoptosis Detection Kit (KeyGen Biotech, Nanjing, China) was used for detecting apoptosis according to the instructions of manufacturer. The Panc-1cells were labeled by Annexin-V and then were detected by a flow cytometer LSR Fortessa (Becton Dickinson).

### Acute toxicological assay

Male BALB/c mice, 4–6 weeks, were purchased from Laboratory Animal Center in Sun Yat-Sen University, Guangzhou, China. These mice were randomly divided into three groups and were then intraperitoneally injected with recombinant proteins at different doses. After two weeks, mice were sacrificed. The organs including heart, liver, spleen, lung, and kidney were fixed in 4% formaldehyde at room temperature for hematoxylin and eosin staining and the blood samples were subjected to the analysis of hepatic or renal functions.

### *In vivo* experiments

All animal procedures were conducted in accordance with the protocols generated by the Institutional Animal Care and Use Committee at the Sun Yat-Sen University. For subcutaneous xenografts, 6-week-old female BALB/c nude mice were injected with 1 × 10^6^ cells in a 100 μl suspension. Then these mice were randomly divided into 2 groups. One group was received the treatment of PTD-RBD-VIF injection and another group was received a control protein PTD-GFP-VIF. These two groups were intraperitoneal injection with 100ug recombinant proteins every three days respectively, until they died.

### Statistics and graphs

Statistical analyses were carried out using Prism software (GraphPad). All of data are reported as mean ± SEM. Differences were found to be significant when P was less than 0.05 or 0.01, as indicated by single (*) or double asterisks (**) within the figures. Most graphs were produced using Prism. Flow cytometry data was processed using FlowJo (Tree Star).

## SUPPLEMENTARY MATERIAL FIGURES



## Abbreviations

HIV-1: human immunodeficiency virus type 1
RBD: Ras binding domain
PTD: protein transduction domain
HECT: Homologous to E6-AP C Terminus
RING: Really Interesting New Gene
E6AP: E6 associated protein
CBL: Casitas B-lineage lymphoma proto-oncogene
CHIP: carboxyl-terminus heat shock cognate 70-interacting protein
VHL: von Hippel-Lindau syndrome
VIF: HIV-1 virion infectivity factor
IPTG: isopropylthio-β-d-galactoside
MFI: mean fluorescent intensity
RNAi: RNA interference
NSCLC: non-small cell lung cancer
BUN: Blood urea nitrogen
CRE: Creatine
AST: Aspartate transaminase
ALT: Alanine transaminase
