# Supplementary material for: A recombinant chimeric protein specifically induces mutant KRAS degradation and potently inhibits pancreatic tumor growth
Source: Oncotarget. 2016 Jun 14;7(28):44299–309. doi: 10.18632/oncotarget.9996 (PMC5190097; doi:10.18632/oncotarget.9996)
Supplement: Supplementary file 1 [file oncotarget-07-44299-s001.pdf]

A recombinant chimeric protein specifically induces mutant KRAS degradation and potently inhibits pancreatic tumor growth

Supplementary Materials

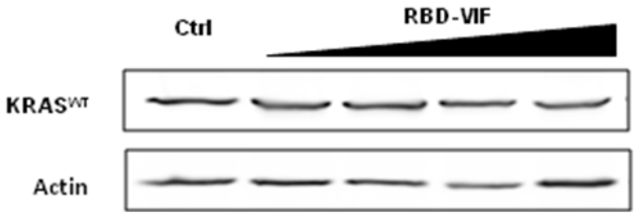

Supplementary Figure S1: Chimeric proteins RBD-VIF has no effect on the degradation of wild-type KRAS. The wild-type KRAS plasmids were co-transfected with different doses of RBD-VIF plasmids into HEK293T cells, and the levels of KRAS were determined by western blotting after 48 h.

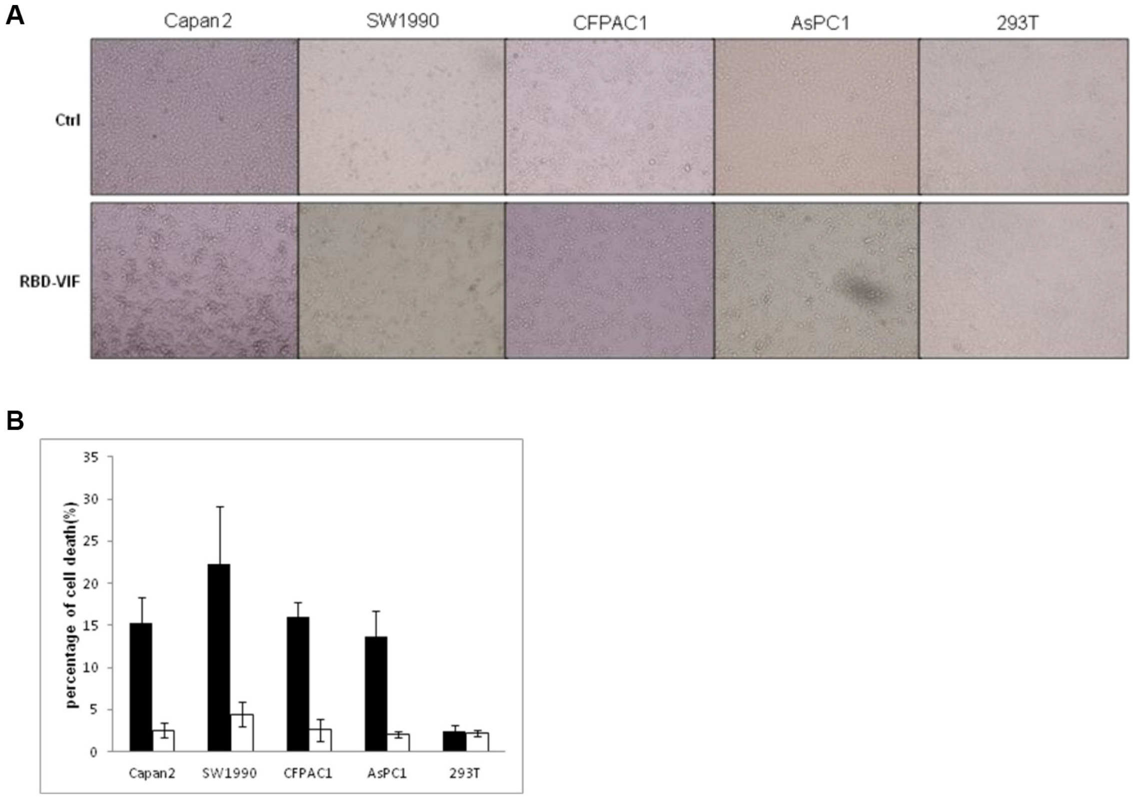

Supplementary Figure S2: Recombinant chimeric proteins induce cell death in many different mutant KRAS-expressing tumor cell lines. (**A**)The purified protein RBD-VIF was added to different cell cultures. After 48 h, cells were examined under microscope. (**B**) The percentage of cell death was evaluated by MTT assay, and mean ± SEM is shown. Error bars indicate SEM.
